# Perihepatic Packing in The Management of Liver Trauma

**DOI:** 10.1155/1991/27272

**Published:** 1991

**Authors:** J. E. J. Krige

**Affiliations:** Department of Surgery University of Cape Town Observatory 7925 South Africa


show that surgery is still the best treatment in many instances in terms of quality of the results, cost and rapid rehabilitation compared to non surgical methods such as interventional radiology or "operative" endoscopy.

P.J. Kestens and J.F. Gigot
Digestive Surgery Department
Medical School Universite Catholique de Louvain
Cliniques Universitaires Saint-Luc
Avenue Hippocrate 10
B–1200 Bruxelles, Belgium

*References*

1. Bismuth, H. and Lazorthes, F. (1981) Les traumatismes operatoires de la voie biliaire principale. Monographie de l'Association Française de Chirurgie (AFC). Masson
2. Hepp, J. (1985) Hepaticojejunostomy using the left biliary trunk for iatrogenic biliary lesions: the French connection. *World J. Surg.* **9**, 507–511
3. Cameron, J.L., Gayler, B.W. and Zuiedma, G.D. (1978) The use of silastic trans-hepatic stents in benign and malignant biliary strictures. *Ann. Surg.* **188**, 552–561

### PERIHEPATIC PACKING IN THE MANAGEMENT OF LIVER TRAUMA

### ABSTRACT

*Hollands, M.J., Little, J.M. (1989) Perihepatic packing: Its role in the management of liver trauma. Aust NZ Surg 59: 21-24.*

**Perihepatic packing was used in 25 of 197 (12.7%) patients presenting with liver trauma to Westmead Hospital over an 8 year period. Packing was used either to provide temporary haemostasis prior to transfer or as part of a definitive treatment plant at this hospital. Thirteen patients were packed prior to transfer. Only two were unstable on arrival, one of whom died. They were compared with 18 'comparison' patients with liver injuries of similar severity. In this group 10 were unstable on arrival ($P = 0.027$), nine of whom died ($P = 0.015$). Packing was used as part of a definitive treatment plan at Westmead on 17 occasions. Four patients were coagulopathic and five had also been packed prior to arrival. Eight of this group died.**

**Packing is a convenient and safe way of controlling major hepatic haemorrhage prior to transfer to a tertiary referral centre. It may also be part of a definitive treatment plan to control hepatic bleeding especially as many patients arrive with a coagulopathy or develop a coagulopathy during the course of surgery to control bleeding. Packing will control haemorrhage until the coagulopathy has been corrected.**

### PAPER DISCUSSION

KEY WORDS: Liver trauma, Hepatic packing.

Haemorrhage can be controlled in the majority of liver injuries by simple methods such as manual compression, suture or accurate discrete ligation of accessible bleeding vessels within the liver wound. The relatively few patients who have major parenchymal or venous injuries, usually following blunt abdominal trauma, require more advanced techniques for control of liver bleeding[1]. During the past decade, the increasing trend in surgical restraint, with fewer major resections for liver trauma has emphasised the role of other lesser procedures for control of bleeding, including hepatotomy or splitting the liver to directly approach deep inaccessible bleeding, porta hepatis inflow control, total hepatic vascular isolation for major venous injuries[2] and the more liberal use of liver packing[3].

In the analysis of data on the role of perihepatic packing in liver trauma, a clear distinction should be made in both the application and duration of liver packing to avoid past problematic semantic differences. The descriptive terms of either resuscitative and therapeutic liver packing are preferable[3]. Resuscitative liver packing implies initial manual compression and packing of the bleeding site after opening the abdomen to control liver blood loss while improving haemodynamic stability and repairing other priority vascular injuries, or while awaiting more experienced surgical assistance. Therapeutic packing, as in the paper under discussion, is used to provide tamponade of liver bleeding and the later retrieval of packs during relaparotomy in theatre after correction of coagulation disorders in an intensive care unit.

The report from the Westmead group is important since few substantial series have critically defined the role of liver packing or provided accurate guidelines for either the indications or application of therapeutic packing in complex liver trauma. A unique feature of the Australian experience is the large number of patients in their series transferred to their unit with packs *in situ* after initial exploration for liver trauma elsewhere. The authors endorse the use of packing during transfer of complex liver injuries by confirming the favourable outcome in their packed group compared to a similar group transferred without the benefit of packing. The reported use of perihepatic packing in the literature varies from 0.5%[4] to 37%[5], the higher figure reflecting referral patterns with the inclusion of patients transferred to trauma centres with packs *in situ*. Moore reported an overall 4% incidence of perihepatic packing in civilian liver injuries treated at eight major North American trauma centres[6]. In a survey of six North American regional trauma referral centres, 25% of patients with major hepatic trauma required packing[7].

The major indication for therapeutic perihepatic packing in Hollands' and Little's paper was transfusion-induced coagulopathy. Thrombocytopaenia, qualitative defects of platelet function and coagulopathy, aggravated by shock, hypothermia and acidosis are common after massive, rapid blood transfusion in major liver trauma[8]. There is consensus that packing is the treatment of choice for transfusion-induced coagulopathy, providing time to return the patient to the intensive care unit safely for further correction of coagulation abnormalities and hypothermia. As in the paper under discussion, others have shown packing to be effective in patients who have coagulopathy with definitive control of bleeding being achieved in up to

80% of patients. Svoboda *et al*[9] reported the specific use of perihepatic packing to treat bleeding secondary to coagulopathy after massive blood transfusion with definitive control of bleeding in 10 of 12 patients. Carmona *et al*[10] compared temporary liver packing in 17 haemodynamically unstable patients with liver trauma refractory to conventional haemostatic techniques to a similar group treated without packing. No patients in the packed group re-bled compared to 3 in the non-packed group, two of whom died of continuing haemorrhage.

The extended role of packing as primary treatment of complex or major hepatic venous injury remains controversial. The authors indicate that all their patients had hepatic venous injuries or multiple hepatic tears. Indeed, the only patient who died after packing failed to control haemorrhage during transfer, had a right hepatic vein injury with avulsion from the inferior vena cava. A body of opinion would, however, advise that an attempt should be made to repair major hepatic venous injury during initial surgery but that packing may be useful as an additional mechanism to control associated coagulopathy and only in select cases should packing be tried as a definitive method of treatment for hepatic venous injury. Other authors have expressed the limitations of packing for control of active bleeding from intrahepatic or retrohepatic veins[11]. There is, however, evidence from individual cases[12] or small series[13] where packing has temporarily controlled juxtahepatic venous bleeding from either vena cava or hepatic veins. We would recommend this manoeuvre only in exceptional circumstances, where the patient is unstable and where further major surgery would be hazardous.

The technique of perihepatic packing is important if effective tamponade and haemostasis is to be assured. Sufficient packs should be inserted to provide adequate external counter pressure. We found that a "six-pack" technique provided the most effective and optimal tamponade[3]. Other authors have recommended interposing either a plastic adhesive drape folded on itself[11], or microfibrillar bovine collagen (Avitene)[9] between the dry laparotomy pads and the underlying liver to avoid clot disruption and to prevent the pack adhering to the exposed hepatic parenchyma during pack removal. An important practical point emphasized by Hollands and Little, is the avoidance of packing into the liver fracture which may provoke bleeding during pack extraction. An attempt should be made to return the liver contour to normal and to re-approximate the edges of the defect by external pressure. A potential complication which may be produced by using an excessive number of packs is an increase in intra-abdominal pressure, caval compression and acute renal failure[11]. The critical pressure limit may be prevented by monitoring the intra-abdominal pressure and not exceeding a pressure of 25 mm Hg[14].

Sepsis is the major source of morbidity following packing. Septic complications ensued in 6 of the 25 patients who had been packed at some time in their treatment in the Westmead series. In a group of New York patients, with predominantly penetrating trauma, the infection rate was significantly increased in packed patients (83%), compared to a similar group (20%) who had debridement-resection without packing[15]. In contrast, a similar analysis from San Francisco reported identical infection rates (29%) in packed and non-packed patients with severe liver injury[10]. Seven of 22 patients who required packing in Cape Town developed local sepsis[3]. We found that patients who had bowel or bile leaks or were packed for more than 72 hours or required repacking, invariably became septic. Current recommendations are to remove the packs within 48 hours after correction of coagulation and metabolic abnormalities and, if possible, to avoid repacking. In liver injuries with gross bowel contamination or bile leaks identified during surgery in whom packing in unavoidable, packs should be retrieved at relaparotomy within 24 hours.

The judicious use of therapeutic perihepatic packing to provide tamponade for control of bleeding in complex liver trauma has evolved as an acceptable and recognized emergency measure over the past decade. The technique does not replace conventional methods of haemostasis and has application in only a small group of select cases with bleeding due to coagulopathy, the transfer of patients from peripheral hospitals to larger centres of expertise or as a life-saving manoeuvre in haemodynamically unstable patients with extensive liver injuries in whom resection would be hazardous. When packing is used, it is important to ensure effective uniform pressure, avoid caval compression, provide ventilatory support and remove packs within 48 hours to minimize the risk of sepsis and to make provision to deal with recurrent bleeding.

Dr. J.E.J. Krige
Department of Surgery
University of Cape Town
Observatory 7925, South Africa

### REFERENCES

1. Terblanche, J, Krige, J.E.J. (1990) Injuries to the liver and bile ducts. In: Acute Abdominal Emergencies. Eds: Williamson, R.C.N., Churchill Livingstone, London.
2. Krige, J.E.J., Worthley, C.S., Terblanche, J. (1990) Severe juxtahepatic venous injury: Survival after prolonged hepatic vascular isolation without shunting. *HPB Surgery*, **3**, (in press).
3. Krige, J.E.J., Bornman, P.C., Terblanche, J. Therapeutic perihepatic packing in complex liver trauma. Br J Surg (in press) tion)
4. Lucas, C.E., Ledgerwood, A.M. (1976) Prospective evaluation of haemostatic techniques for liver injuries. *J. Trauma*, **16**, 442-451.
5. Calne, R.Y., Wells, F.C., Forty, J. (1982) Twenty-six cases of liver trauma. *Br. J. Surg.*, **69**, 365-368.
6. Moore, E.E. (1984) Critical decisions in the management of hepatic trauma. *Am. J. Surg.*, **148**, 712-716.
7. Cogbill, T.H., Moore, E.E., Jurkovich, G.J., Feliciano, D.V., Morris, J.A., Mucha, P. (1988) Severe hepatic trauma: a multicenter experience with 1335 liver injuries. *J. Trauma*, **28**, 1433-1438.
8. Clagett, G.P., Olsen, W.R. (1978) Non-mechanical haemorrhage in severe liver injury. *Ann. Surg.*, **187**, 369-374.
9. Svoboda, J.A., Peter, E.T., Dang, C.V., Parks, S.N., Ellyson, J.H. (1982) Severe liver trauma in the face of coagulopathy. A case for temporary packing and early re-exploration. *Am. J. Surg.*, **144**, 717-721.
10. Carmono, R.H., Peck, D.Z., Lim, R.C. (1984) The role of packing in planned re-operation in severe hepatic trauma. *J. Trauma*, **24**, 779-784.
11. Feliciano, D.V., Mattox, K.L., Burch, J.M., Bitondo, C.G., Jordan, G.L. (1986) Packing for control of hepatic haemorrhage. *J. Trauma*, **26**, 738-743.
12. Ferguson, C.M. (1989) Hepatic packing in major liver trauma. *Arch. Surg.*, **124**, 508-509.
13. Calne, R.Y., McMaster, P., Pentlow, B.D. (1979) The treatment of major liver trauma by primary packing with transfer of the patient for definitive treatment. *Br. J. Surg.*, **66**, 338-339.
14. Kron, I.L., Harmon, P.K., Nolan, S.P. (1984) The measurement of antra-abdominal pressure as a criterion for abdominal re-exploration. *Ann. Surg.*, **199**, 28-30.
15. Ivatury, R.R., Nallathambi, M., Gundoz, T. *et al.* (1986) Liver packing for uncontrolled haemorrhage. A reappraisal. *J. Trauma*, **26**, 744-753.

